# Establishment of an imaging-based screening pipeline for the identification of human ribosome biogenesis inhibitors

**DOI:** 10.1186/s12915-025-02425-2

**Published:** 2025-10-21

**Authors:** Claudia Gafko, Réka Hollandi, Kerstin Dörner, Matteo Rosellini, Ivo Zemp, Peter Horvath, Ulrike Kutay

**Affiliations:** 1 Institute of Biochemistry, Department of Biology, ETH Zurich, Zurich, 8093 Switzerland; 2 Molecular Life Sciences Ph.D. Program, Zurich, 8057 Switzerland; 3 Synthetic and Systems Biology Unit, Biological Research Center, Szeged, Hungary; 4 Present Address: Biozentrum, University of Basel, Basel, Switzerland

**Keywords:** Chemical compound screen, Ribosome synthesis, High-content screening, Microscopy, Cancer, Drug, Ribosomal subunit, rRNA, Image analysis, Machine learning

## Abstract

**Background:**

Ribosomes are huge ribonucleoprotein particles that mediate protein synthesis in all organisms. The synthesis of ribosomes is a complex process that involves hundreds of supporting factors in mammalian cells, including proto-oncogenes and tumor suppressors. Dysregulation of ribosome biogenesis can contribute to tumorigenesis, and the increased production of ribosomes in cancer cells is known to promote proliferative cell growth. Therefore, ribosome biogenesis represents an attractive vulnerability of cancer cells that ought to be exploited for the development of anti-cancer drugs. Despite the large number of trans-acting factors promoting ribosome assembly including potentially druggable enzymes, only few chemical inhibitors that act on ribosome biogenesis, especially downstream of pre-rRNA transcription, have been identified to date.

**Results:**

To enable large-scale screens for chemical compounds that interfere with ribosome biogenesis, we have established a pipeline to perform single-cell, imaging-based screening campaigns using four different readouts, including fluorescent ribosomal protein reporters (RPS2-YFP, RPL29-GFP) and immunofluorescence analyses of the ribosome biogenesis factor ENP1(BYSL), in HeLa cells, a human cancer line. We have assessed the robustness of our high-content screening approach by performing a pilot screen using a library comprising more than 1000 FDA-approved drugs with known targets in other pathways. This pilot screen obtained excellent quality scores and identified ten compounds as hits. These hit compounds likely affect ribosome synthesis indirectly, the majority by inducing DNA damage or by inhibiting the proteasome. We therefore used the identified compounds to establish appropriate counter assays for DNA damage and proteasome inhibition, to exclude common indirect effects in the downstream analysis of such screening campaigns.

**Conclusions:**

The established screening pipelines provide a robust, efficient, and sensitive experimental framework to identify chemical compounds that impair ribosome synthesis. The combination of readouts allows to distinguish effects on pre-rRNA synthesis from downstream effects on ribosome assembly. Established counter assays on DNA damage and protein degradation enable to exclude effects on these pathways, which commonly interfere with ribosome synthesis indirectly. The developed assays are easily scalable to screen libraries of higher complexity in the future.

**Supplementary Information:**

The online version contains supplementary material available at 10.1186/s12915-025-02425-2.

## Background

It was noticed more than a century ago that enlarged nucleolar size and number are hallmarks of cancer cells [1]. By now, it is well established that these changes are associated with the hyperactivation of ribosomal DNA transcription upon cellular transformation [2, 3]. The analysis of nucleolar morphology is commonly employed as a diagnostic biomarker of cancerous cells, assessment of which is increasingly being supported by artificial intelligence pipelines [4, 5]. While it was initially assumed that ribosome biogenesis is merely upregulated in cancer cells to facilitate their increased proliferative growth, later studies revealed an intricate crosstalk between ribosome production and critical tumor suppressors (e.g., p53) or proto-oncogenes (e.g., c-Myc) that can even contribute to malignant transformation [6–12].

Many anti-cancer therapies rely on DNA-reactive agents or topoisomerase inhibitors, which function by damaging DNA or hindering DNA synthesis. In proliferating cells, DNA damage activates DNA repair and cell cycle checkpoints, thereby preventing apoptotic cell death. However, in cancer cells, these mechanisms often function poorly, and DNA damaging agents induce apoptosis [13–15]. Interestingly, these anti-cancer drugs act not only on DNA, but also frequently inhibit ribosome biogenesis [16, 17]. For example, oxaliplatin, doxorubicin, or methotrexate all inhibit precursor ribosomal RNA (pre-rRNA) transcription [16, 18, 19], although it is currently unclear whether inhibition of ribosome synthesis contributes to their therapeutic effects.

Eukaryotic ribosome biogenesis is a complex process that starts with the synthesis of a long polycistronic rRNA precursor (47S in humans) by RNA polymerase I (RNAPI) in the nucleolus. The nascent primary transcript associates with a subset of ribosomal proteins (RPs) and various ribosome biogenesis factors to form an early ribosomal precursor particle, referred to as the small subunit (SSU) processome. After modification and trimming of the pre-ribosomal RNA (pre-rRNA), the precursors of 40S and 60S ribosomal subunits emerge by pre-rRNA cleavage within the SSU processome, and both subunits then further mature independently. Remodeling and maturation of these pre-ribosomal particles is accompanied by incorporation of additional RPs as well as the endo- and exonucleolytic trimming of the pre-rRNA. After export to the cytoplasm, final maturation steps take place, yielding translation-competent 40S and 60S subunits. This intricate assembly process requires the coordinated action of more than 200 ribosome biogenesis factors, which collectively support the processing, modification, and folding of pre-rRNA along with the incorporation of RPs [20–22]. In mammalian cells, activation of the tumor suppressor p53 is triggered by perturbation of ribosome synthesis in the nucleolus, a condition referred to as “nucleolar stress” [23–25]. Therefore, perturbation of early, nucleolar steps of the ribosome synthesis pathway may represent an attractive therapeutic opportunity, especially for p53-positive cancers [26].

While it might be expected that ribosome biogenesis inhibitors would indistinctively target healthy and neoplastic cells, it has in fact been demonstrated that these inhibitors exhibit a selective cytotoxicity for cancer cells due to their enhanced rRNA synthesis rate [26, 27]. The notion that the ribosome biogenesis pathway is vulnerable to chemotherapeutic intervention is strongly supported by the clinical successes of CX-5461 [28–31] and BMH-21 [32, 33]. Both drugs effectively impair ribosome biogenesis by disruption of RNAPI-mediated transcription. CX-5461 interferes with binding of the SL1 complex to the rDNA promoter, thereby disrupting the assembly of the RNAPI transcription initiation complex [28], but it also evokes DNA damage [34, 35]. BMH-21 intercalates into GC-rich rDNA, thereby preventing rRNA transcription [36], and traps topoisomerase II on chromatin [37].

Even though human ribosome biogenesis involves a plethora of potentially druggable enzymes [20], the vast majority of identified inhibitors interfere with the pathway at the level of rRNA transcription or rather indirectly via DNA damage. Specific inhibitors that act on nucleolar steps of ribosome synthesis with the capacity to elicit p53 induction, yet lack the undesired side effect of DNA damaging agents, remain to be identified. Studies in yeasts have described a number of compounds that block 60S biogenesis, including diazaborine, which blocks the AAA-ATPase Drg1 [38], Rbin-1, which inhibits the AAA-ATPase Rea1/Midasin, and, recently, usnic acid, a lichen secondary metabolite that inhibits a nucleolar step of 60S subunit maturation by an as-of-yet undefined mechanism [39]. Whether any of these compounds inhibits mammalian ribosome synthesis is currently unclear. On the other hand, direct inhibitors of human ribosome biogenesis have only recently started to be identified, and direct inhibitors of nuclear steps of this process are still lacking. Some promising compounds target RIOK2 [40–44], a biogenesis factor that promotes cytoplasmic maturation of 40S ribosomal subunits [45]. Furthermore, a high-throughput drug screen in human cells using a constitutively expressed RPS9 reporter fused to the HaloTag epitope identified novel inhibitors of rRNA synthesis, although their targets remain to be identified [46, 47].

Most cancer cells are genetically unstable and can rapidly acquire resistance to selected treatments, substantiating the need for further discovery and development of novel inhibitors. Since ribosome biogenesis has emerged as an exceptionally promising target for chemotherapeutical interventions, and several established anti-cancer agents were shown to inhibit ribosome biogenesis at different stages [16, 17], we set out to establish a screening pipeline for human ribosome synthesis inhibitors. We have implemented an imaging-based chemical compound screening workflow to identify inhibitors of the ribosome biogenesis pathway in human cancer cells and successfully tested the pipeline using a library comprising more than 1000 FDA-approved drugs.

## Results

### Development of a single-cell, imaging-based workflow for screening of ribosome biogenesis inhibitors in HeLa cells

**Fig. 1:**
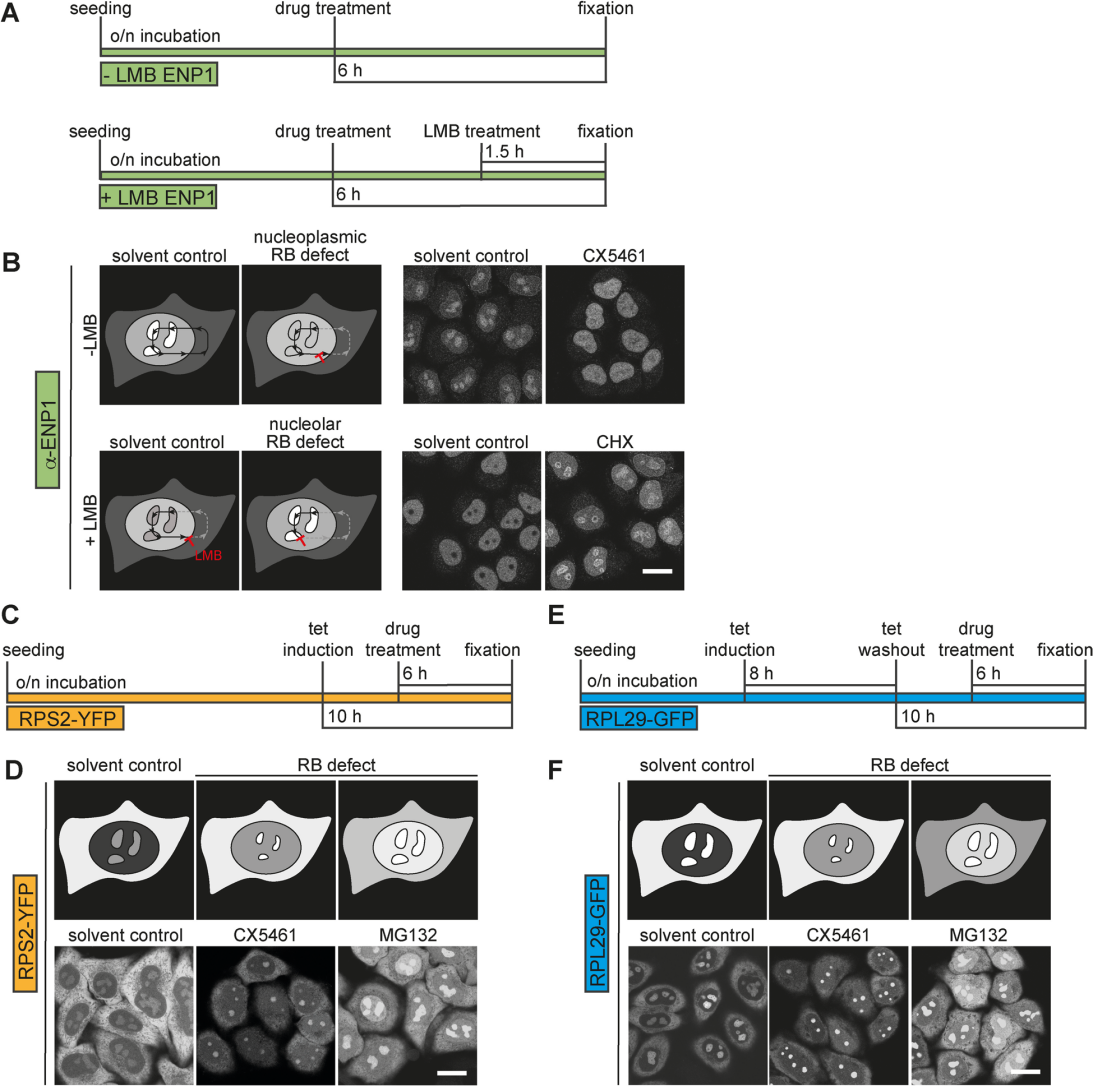
Overview of the experimental approach and the four microscopic readouts used for chemical compound screening on ribosome biogenesis. **A** Timeline of the screening assay with ENP1 immunofluorescence in the absence or presence of 10 nM leptomycin B (LMB) as readouts (− LMB; + LMB). **B** Schematic overview and representative images of ENP1 immunofluorescence of control HeLa K cells and cells treated with the indicated compounds (2 nM CX-5461; 100 µg/ml CHX) for 6 h, in the absence or presence of the nuclear export inhibitor LMB. Red icons indicate at which steps ribosome synthesis is visibly inhibited. Scale bar: 20 µm. **C** Timeline of the screening assay for the RPS2-YFP reporter cell line. **D** Schematic overview and example images of the RPS2-YFP reporter. Reporter expression was induced with 0.5 µg/ml tetracycline for 10 h. Cells were treated with the indicated drugs (2 nM CX-5461; 10 µM MG132) 6 h before fixation. Scale bar: 20 µm. **E** Timeline of the screening assay for the RPL29-GFP reporter cell line. **F** Schematic overview and example images of the RPL29-GFP reporter. Reporter expression was induced with 0.5 µg/ml tetracycline for 8 h, followed by incubation for 10 h in tetracycline-free medium. Cells were treated with library drugs 6 h before fixation. Scale bar: 20 µm

To visualize defects in ribosome biogenesis induced by small molecule inhibitors, we devised a high-throughput, imaging-based, chemical screening workflow in HeLa cells, adopting a suite of microscopic assays that we have previously used to study mechanistic aspects of human ribosome synthesis by RNA interference (RNAi) [48–50]. To quantitatively score the effects of chemical compounds on the maturation of ribosomal subunits, we altogether applied four different readouts, adjusting the assays to be compatible with the screening of small molecule inhibitors, which act much more rapidly than siRNAs. We chose a drug treatment time of 6 h before cell fixation (Fig. 1), giving sufficient time for compound-induced phenotypes to be robustly established in our assays.

As the first readout, we chose immunofluorescence of the shuttling 40S ribosome biogenesis factor ENP1/BYSL [50] (Fig. 1A, B). At steady state, ENP1 is localized in the cell nucleus where it is enriched in nucleoli. ENP1 joins pre-40S particles during the nucleolar formation of the SSU processome [51] and accompanies pre-40S particles to the cytoplasm, where it is dissociated and quickly recycled to the nucleus [45, 48, 50]. Since nucleolar size and maintenance of nucleolar integrity depend on the synthesis of pre-rRNA by RNAPI [52–55], the nucleolar steady-state localization of ENP1 can be exploited to visualize changes in rDNA transcription. Inhibition of RNAPI activity, as for instance induced by CX-5461 [28, 29], leads to nucleolar disintegration, loss of nucleolar ENP1 staining, and its dispersal throughout the nucleoplasm (Fig. 1B).

ENP1 immunofluorescence in the presence of the CRM1/XPO1 inhibitor leptomycin B (LMB) was used as a second readout to detect defects in the nucleolar assembly of pre-40S subunits (Fig. 1A, B). When nuclear export of pre-40S subunits, which is dependent on CRM1/XPO1, is blocked by LMB [56], ENP1 relocalizes from nucleoli to the nucleoplasm (Fig. 1B), where it accumulates as part of newly made pre-40S subunits that fail to exit the nucleus [45]. However, when nucleolar steps of pre-40S assembly are defective, pre-40S subunits cannot leave nucleoli, manifesting in nucleolar retention of ENP1 in the presence of LMB [48]. Nucleolar retention of ENP1 is, for instance, induced by the inhibition of protein synthesis by cycloheximide (CHX) [57], which blocks the production of RPs and thereby impairs early steps in subunit assembly (Fig. 1B). Thus, visualizing changes in ENP1 localization enables the detection of defects in both pre-rRNA transcription (in the absence of LMB) and nucleolar pre-40S assembly defects (in the presence of LMB) (Fig. 1A, B).

As the third readout, we used a previously established HeLa reporter cell line for 40S subunit biogenesis, which expresses a tetracycline-inducible RPS2-yellow fluorescent protein (RPS2-YFP) fusion [48, 50]. We induced the expression of RPS2-YFP for 10 h (Fig. 1C). During this period, RPS2-YFP is produced, incorporated into ribosomal pre-particles in the nucleolus, and subsequently exported to the cytoplasm as part of maturing 40S subunits. In control cells, RPS2-YFP localizes to the cytoplasm and nucleoli. Drug-induced inhibition of nucleolar or nucleoplasmic steps of 40S maturation, e.g., induced by treatment with CX-5461 or the proteasome inhibitor MG132 [58], results in reduced levels of RPS2-YFP in the cytoplasm and its accumulation in nucleoli and/or the nucleoplasm (Fig. 1C, D).

Similarly, to monitor defects in pre-60S ribosomal subunit maturation, we used a HeLa cell line expressing a tetracycline-inducible RPL29-green fluorescent protein (RPL29-GFP) reporter construct as our fourth readout [49, 50]. RPL29-GFP is expressed for 8 h followed by a chase period of 10 h in medium without tetracycline. During this time, RPL29-GFP is first synthesized, imported into the nucleus, assembled into pre-60S particles in the nucleolus, and then exported back to the cytoplasm as part of maturing 60S subunits. Akin to the RPS2-YFP reporter, drug-induced perturbations of nucleolar or nucleoplasmic steps of 60S subunit maturation result in a loss of cytoplasmic signal and stronger accumulation of RPL29-GFP in the nucleus (Fig. 1E, F).

**Fig. 2:**
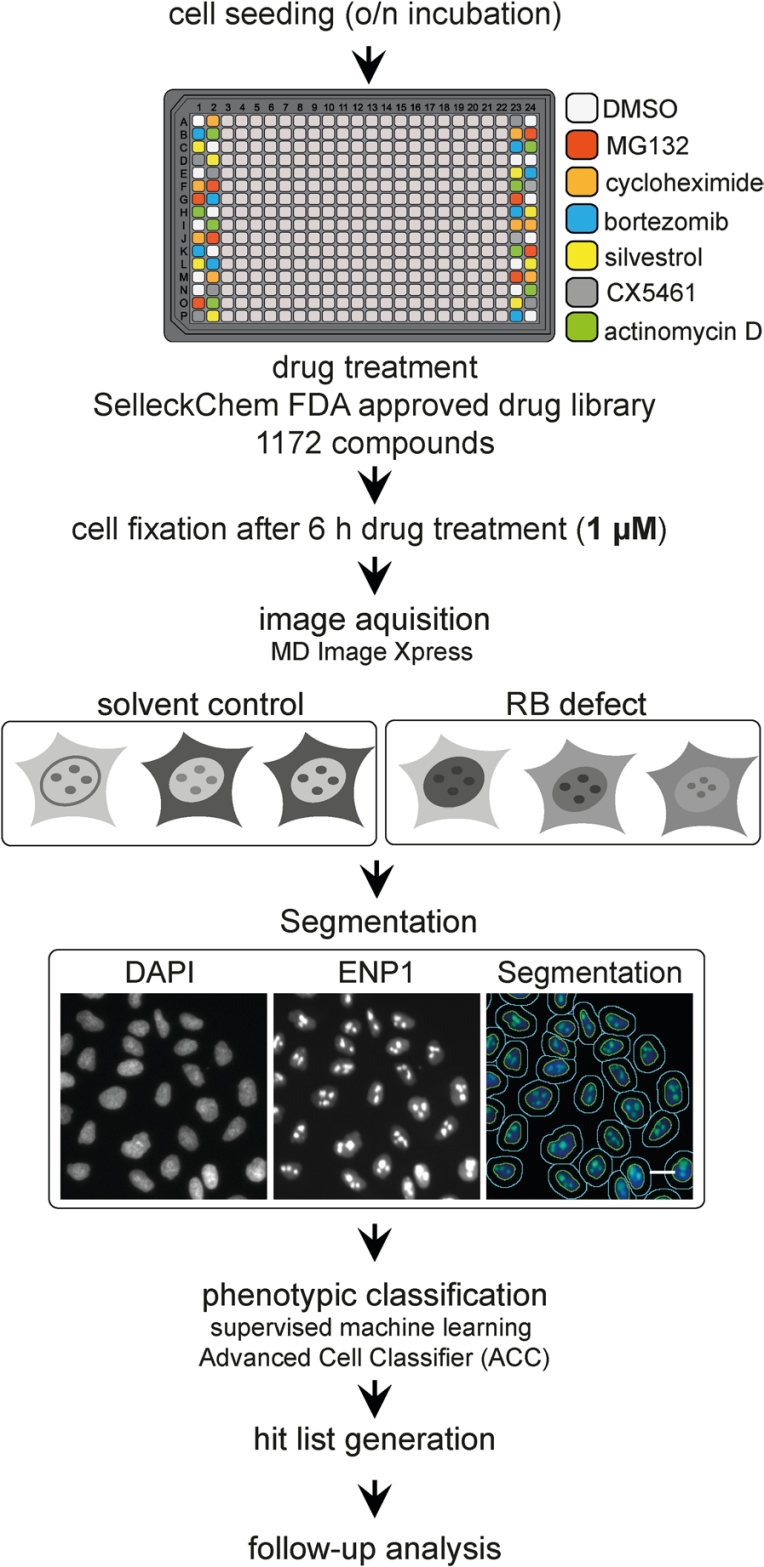
Schematic representation of the screening campaign setup to identify inhibitors of ribosome biogenesis. Cells were seeded into 384-well plates 24 h before their treatment with 1 µM of each of the 1172 chemical compounds contained in the SelleckChem FDA-approved drug library. Reporter expression in the RPS2-YFP and RPL29-GFP cell lines was induced as described in Fig. 1. MG132 (10 µM), CHX (100 µg/ml), bortezomib (2 µM), silvestrol (1 µM), CX-5461 (2 nM), and ActD (10 nM) were used as positive controls. After 6 h of drug treatment, cells were fixed, subjected to immunofluorescence for the ENP1 readouts, and imaged by automated image acquisition. Images were illumination-corrected, segmented (see example segmentation of the screening data, scale bar 20 µm), and subjected to phenotypic hit classification using the Advanced Cell Classifier (ACC), a software package for supervised machine learning [62]. Compounds classified as hits were subsequently validated

Due to the current lack of direct inhibitors of nuclear steps of human ribosome biogenesis, we decided to exploit compounds known to indirectly affect ribosome biogenesis as positive controls in our assays, such as the aforementioned inhibitors of protein degradation (MG132), mRNA translation (CHX), or rDNA transcription (CX-5461) (Fig. 2A). In addition, we chose a second inhibitor for each of the three pathways, namely (1) the proteasome inhibitor bortezomib [59], (2) silvestrol, an inhibitor of translation that targets the initiation factor eIF4A [60], and (3) the well-established RNAPI inhibitor actinomycin D (ActD) [61]. As expected, bortezomib and silvestrol treatment, like MG132 and CHX, respectively, resulted in the retention of ENP1 in nucleoli (in the presence of LMB) as well as in the nuclear accumulation of the RPS2-YFP and RPL29-GFP reporters (Fig. 1, Fig. S1). Perturbation of rDNA transcription by treatment with ActD, like CX-5461, resulted in nucleoplasmic dispersal of ENP1 staining and smaller nucleoli, as detected by the fluorescent RP reporters (Fig. S1).

### Screening of an FDA-approved compound library confirms the robustness and sensitivity of the screening assays

To assess the robustness and sensitivity of the screening assays, we performed pilot screens with all four readouts using an FDA-approved drug library comprising 1172 compounds (SelleckChem). Expected benefits of this library were the structural diversity, confirmed bioactivity, and cell permeability of its compounds. Furthermore, we reasoned that knowledge of the molecular targets of the individual drugs would allow rationalizing how identified hits may affect ribosome biogenesis.

**Fig. 3:**
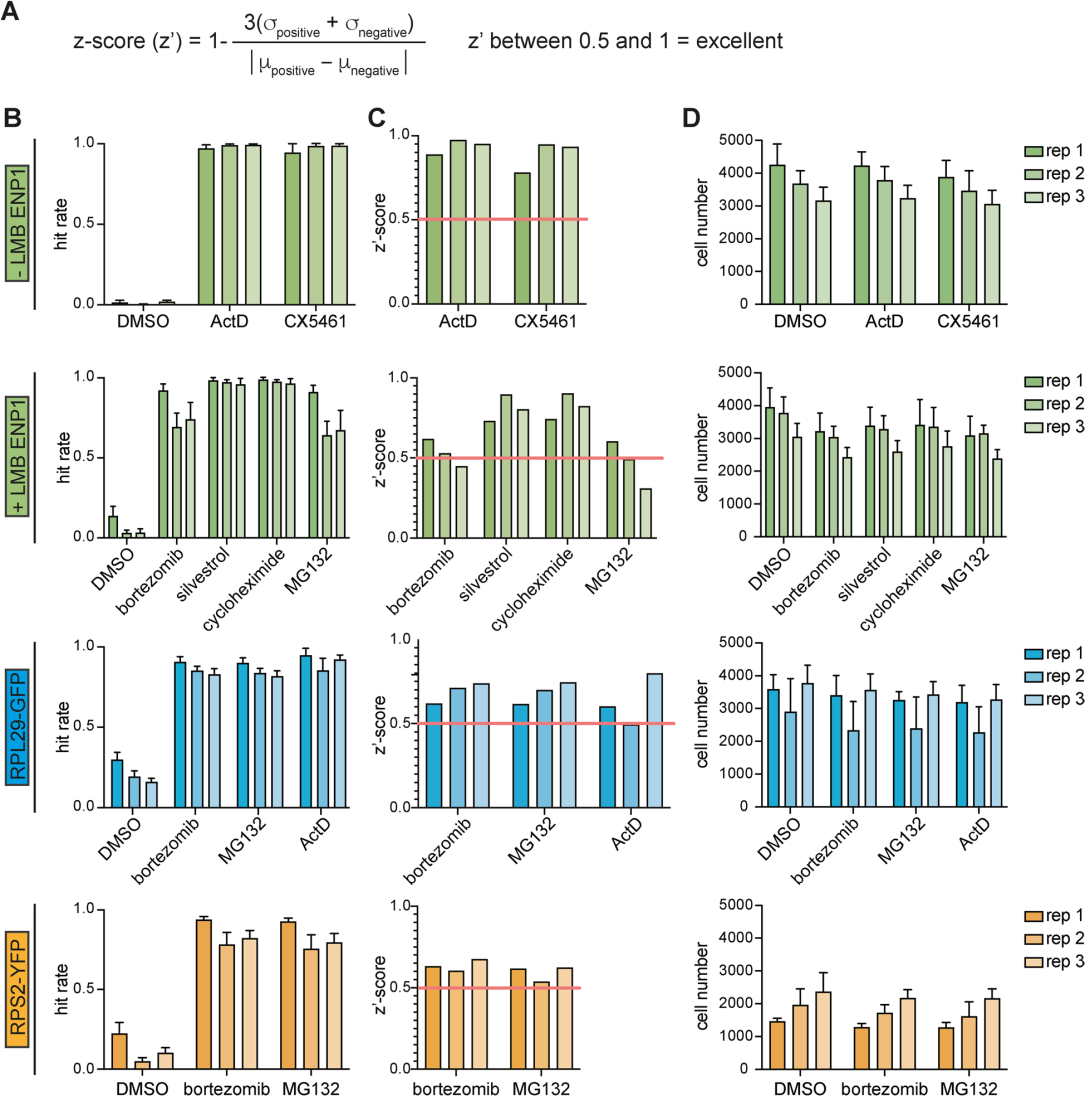
Hit rates and z′ scores confirm the robustness of the screening assays. **A** Formula to calculate z′. A z-score (z′) is a statistical measure to validate the quality of high-throughput screening assays. σ: standard deviation, μ: mean. **B** Hit rates of each biological replicate of the pilot screens. Hit rates were defined as the percentage of cells with nucleoplasmic localization of ENP1 over all interphase cells (for − LMB ENP1), with nucleolar accumulation of ENP1 over all interphase cells (for + LMB ENP1) or the percentage of cells with nuclear localization of RPS2-YFP or RPL29-GFP over all reporter-positive interphase cells. Data depicts mean hit rate per condition within a replicate + SD. All experiments were performed in biological triplicates. **C** z′ scores for the positive controls of all biological replicates used in the pilot screens compared to the DMSO control. **D** Total cell numbers of the three biological replicates. Data depicts the mean cell number per condition within a replicate + SD

The pilot screens with the four established microscopic readouts were performed in biological triplicates in 384-well plates (Fig. 2, Table S1). In the screening pipeline that we have devised, cells were seeded, induced with tetracycline according to their established timelines in the case of the reporter cell lines (Fig. 1A, C, E), and then treated with 1 µM of the library compounds for 6 h before cell fixation. We used the compounds at 1 µM to limit the identification of inhibitors of low potency. Then, after immunofluorescence staining for the ENP1 readouts, cells were subjected to automated fluorescence microscopy and image analysis. Quantification of the screening data relied on an image segmentation and machine learning pipeline that we had successfully used in the past for the analyses of high-content RNAi screens on ribosome biogenesis [48–50]. In essence, images were first illumination-corrected and then segmented based on Hoechst staining of cell nuclei using a customized version of CellProfiler [63]. Phenotypic hit classification was performed by supervised machine learning with the Advanced Cell Classifier [62]. To calculate hit rates, individual cells were assigned into predefined phenotypic classes. These phenotypic classes included cells displaying predominantly nucleolar, nucleoplasmic, or cytoplasmic signals for the respective readouts. Cells that did not display a fluorescent signal or were classified as mitotic, apoptotic, or incorrectly segmented were not considered for hit rate calculations. For each of the four readouts, we defined cells as hits if they fell into predefined phenotypic classes distinct from solvent-treated control cells (DMSO). For example, for the ENP1 readout without LMB treatment, the hit rate was defined as the percentage of cells with nucleoplasmic ENP1 signal over all cells with either nucleoplasmic or nucleolar ENP1 (Fig. 3B). Similarly, the hit rates for the RPS2-YFP or RPL29-GFP reporter cell lines were defined as the fraction of reporter-positive cells showing reduced cytoplasmic RPL29-GFP or RPS2-YFP localization accompanied by nucleolar and/or nucleoplasmic accumulation of the RP reporters, over all reporter-positive interphase cells.

The quality and robustness of our screening campaign was assessed by statistical analyses and calculation of z′ scores for the respective readouts (Fig. 3A, C; Table S2). The z′ score provides a numerical quality measure for high-throughput screening assays [64]. A high z′ score is obtained when the means of the negative and positive controls significantly differ from each other and the standard deviations of both controls are low. An assay is considered excellent when the z′ score is between 0.5 and 1 [64]. We indeed obtained excellent z′ scores for all four microscopic readouts, almost all of which were larger than 0.5, confirming the robust performance of our assays (Fig. 3C). Calculations of cell numbers over all replicates confirmed that these values were similar across replicates of the same readout and sufficient for the subsequent analysis of hits (Fig. 3D).

### The screening campaign identifies 10 hit compounds

**Fig. 4:**
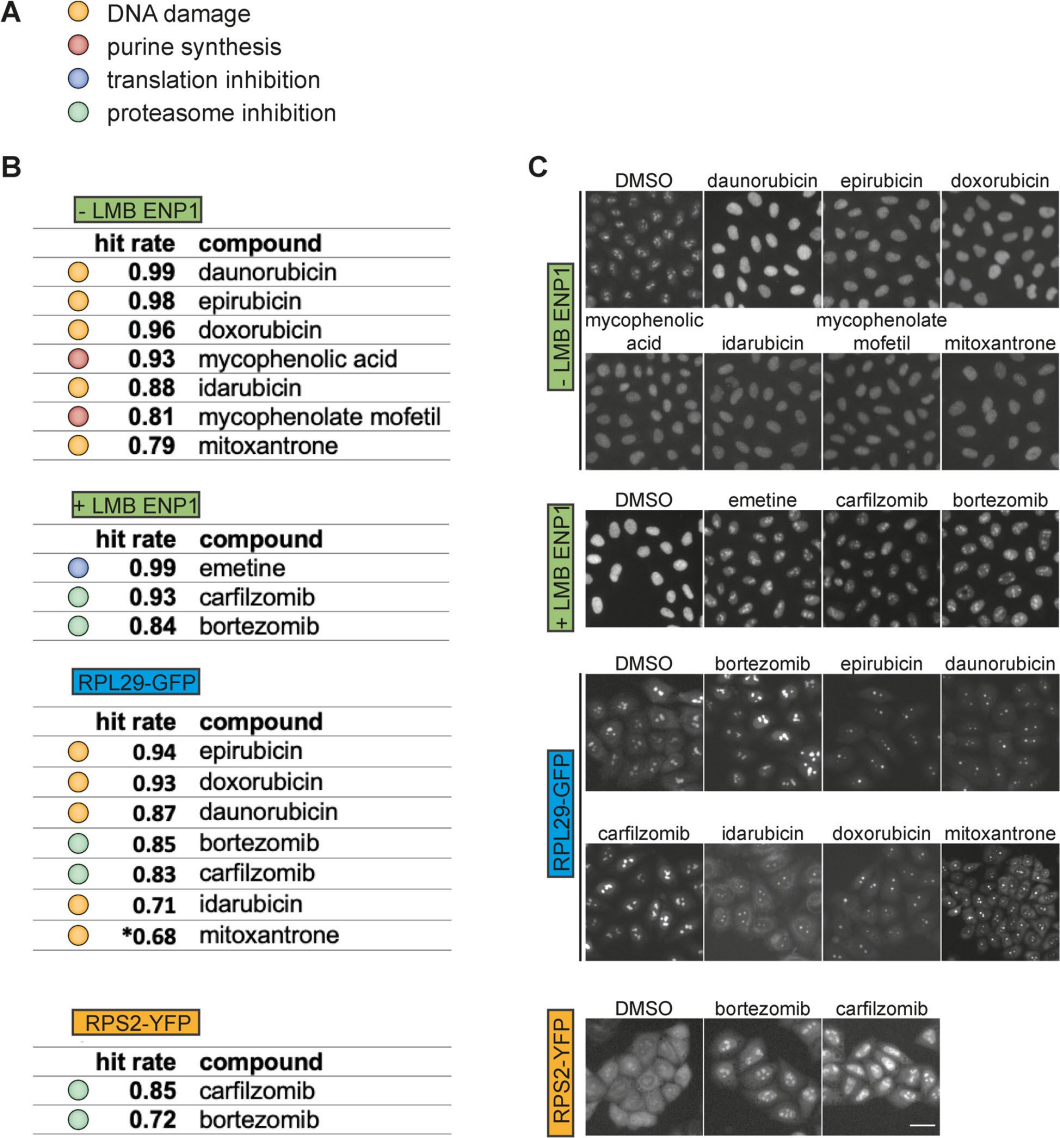
The pilot screening campaign identified 10 hit compounds. **A** Color code for the four biological categories into which the 10 hit compounds were assigned. **B** Calculated hit rates of library compounds for each of the microscopic readouts. Data depicts the mean hit rates of the three biological replicates per readout. *Mitoxantrone was identified as a hit only in replicates 1 and 3. **C** Example images of hit compounds identified in the pilot screens (quantification of the respective hit rates is shown in **B**). Scale bar: 20 µm

For each of the readouts, we next generated a hit list by applying a stringent cutoff of five standard deviations from the mean false-positive rate of the DMSO negative control, followed by visual confirmation of the raw imaging data for the putative hits. Collectively, we identified ten “hit” compounds across the four microscopic readouts. As the molecular targets for most of the drugs contained in the SelleckChem FDA-approved drug library are known, we could assign each of the ten compounds into one of four functional categories, namely (1) DNA damage-inducing agents (doxorubicin, daunorubicin, idarubicin, epirubicin, and mitoxantrone), (2) purine synthesis inhibitors (mycophenolic acid and mycophenolate mofetil), (3) protein degradation inhibitors (bortezomib (also present in the compound library) and carfilzomib), and (4) the translation inhibitor emetine (Fig. 4A). The compounds obtained high hit rates (Fig. 4B), and the induced phenotypes included nucleoplasmic accumulation of ENP1 in the ENP1 staining without LMB treatment (− LMB ENP1), nucleolar retention of ENP1 in the presence of LMB (+ LMB ENP1), and nucleolar and/or nucleoplasmic accumulation of the RP reporters (RPL29-GFP, RPS2-YFP) as well as a decrease in the size of nucleoli, best observed with RPL29-GFP (Fig. 4C, example images of the screening data).

The DNA damage-inducing drugs doxorubicin, daunorubicin, epirubicin, idarubicin, and mitoxantrone all caused nucleoplasmic accumulation of ENP1 (in the absence of LMB) and a reduction in nucleolar size (visible in the RPL29-GFP panels). The anthracycline doxorubicin and its derivatives epirubicin, daunorubicin, and idarubicin as well as mitoxantrone are known to interact with DNA by intercalation and binding to DNA-associated enzymes. More specifically, they inhibit the progression of topoisomerase II thereby preventing DNA replication, which consequently leads to DNA damage and cytotoxic effects [65–67].

Also, mycophenolic acid and mycophenolate mofetil induced a nucleoplasmic accumulation of ENP1 in the absence of LMB. These two immunosuppressant drugs act by reversible and non-competitive inhibition of inosine monophosphate dehydrogenase (IMPDH), which is an essential enzyme for de novo synthesis of guanine nucleotides [68]. Since nucleotides are the main building blocks for the biosynthesis of DNA and RNA, inhibition of guanine nucleotide synthesis may not only affect DNA replication but also inhibit the biogenesis of ribosomes more directly via a shortage of GTP that is needed for pre-rRNA transcription. Indeed, when pre-rRNA synthesis and processing were analyzed by Northern blotting using a probe (5′ ITS1) that detects all intermediates of pre-18S rRNA maturation, both mycophenolic acid and mycophenolate mofetil inhibited 47S pre-rRNA production (Fig. S2). This was accompanied by the disappearance of the downstream processing intermediates, consistent with the proposed effect of mycophenolate acid on rRNA production [69, 70].

Akin to inhibition by CHX and silvestrol, the protein synthesis inhibitor emetine caused nucleolar retention of ENP1 in the presence of LMB (Fig. 4C), likely because ribosome biogenesis is stalled during the early nucleolar assembly steps when newly synthesized RPs are lacking. Emetine binds to the ribosomal E-site on the 40S subunit, thereby inhibiting mRNA/tRNA translocation [71, 72].

Lastly, we identified the proteasome inhibitors carfilzomib and bortezomib as hits, the latter of which also served as a positive control during our screening campaign. While the boron atom of bortezomib reversibly binds and inhibits the catalytic sites of the proteases within the proteasome [59, 73, 74], carfilzomib, a modified epoxyketone, exhibits a structurally and mechanistically distinct mode of action. It irreversibly and selectively inhibits the chymotrypsin-like activity of the proteasome [75, 76]. Treatment with bortezomib or carfilzomib led to nucleolar accumulation of ENP1 in the + LMB ENP1 readout. For the fluorescent RP reporters, these compounds resulted in a reduction of the cytoplasmic signal accompanied by a strong nucleolar/nucleoplasmic accumulation (Fig. 4C).

### Counter assays for DNA damage or proteasomal inhibition

Ribosome biogenesis is highly interconnected with multiple other cellular pathways. For instance, the sensitivity of ribosome production to DNA damage is well established [77, 78]. Furthermore, translation factors and components of the ubiquitin–proteasome system had scored highly in previous RNAi screens for components required for ribosome production [48–50]. Since our chemical screening assay shall be used in the future to identify small molecules that directly interfere with the ribosome biogenesis pathway, we next implemented counter assays to rule out that identified hit compounds act indirectly by inducing DNA damage or by inhibiting the proteasome.

**Fig. 5:**
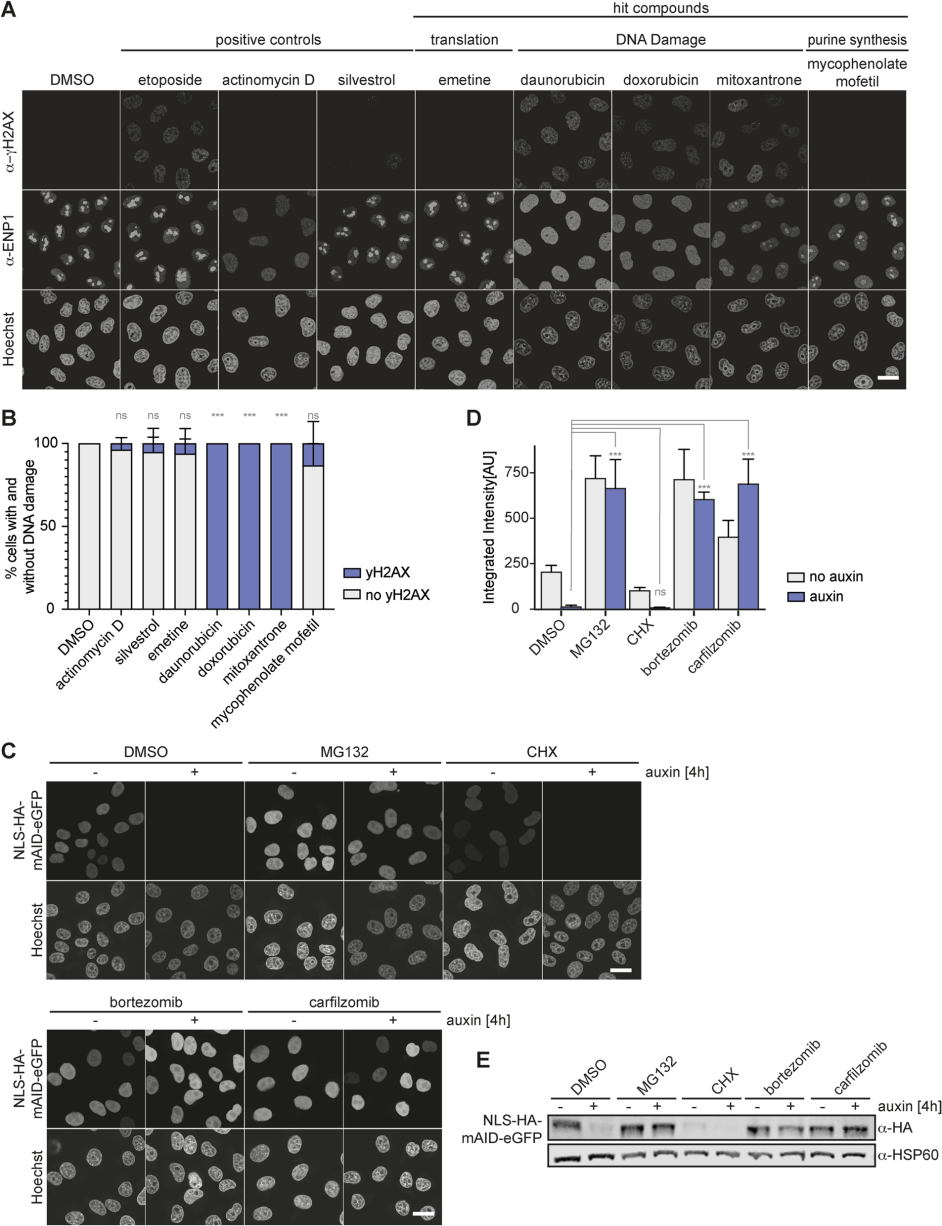
Counter assays for DNA damage and the inhibition of protein degradation. **A** Analysis of DNA damage. HeLa K cells were treated with 1 µM of selected hit compounds or with 10 µM of etoposide (positive control for DNA damage), 10 nM of ActD (positive control for inhibition of rDNA transcription), or 1 µM silvestrol (positive control for inhibition of translation) for 6 h. Cells were fixed and subjected to immunofluorescence analysis of ENP1 and the DNA damage marker γH2AX, which confirmed that daunorubicin, doxorubicin, and mitoxantrone elicited a DNA damage response, as expected. Scale bar: 20 µM. **B** Quantification of cells in **A** stained with an γH2AX antibody (proportion of γH2AX-positive cells over all analyzed cells identified based on Hoechst staining, manual counting). Mean + SD, *N* = 3 biological replicates. Number of cells per condition: *n* > 66, ***p ≤ 0.001, two-sided Student’s *t*-test. **C** Analysis of inhibition of protein degradation. HeLa FRT TetR OsTIR1-9myc cells were induced with tetracycline (0.5 µg/ml, 24 h) to express HA-mAID-NLS-eGFP. Cells were then treated for 6 h with 1 µM of the indicated hit compounds. Degradation of mAID-tagged eGFP was induced by 4-h treatment with auxin (500 µM). Confocal microscopy of the eGFP signal confirmed that the compounds MG132, bortezomib, and carfilzomib inhibited protein degradation, as expected. Scale bar: 20 µm. **D** Quantification of cells in **C** expressing HA-mAID-NLS-eGFP. Mean + SD, *N* = 3, *n* > 70, ***p ≤ 0.001, ns *p* > 0.05, two-sided Student’s *t*-test. **E** Western blot analysis of experiment in **C** showed sustained GFP signal in cells treated with MG132, carfilzomib, and bortezomib after auxin addition, which confirmed inhibition of the proteasome after treatment with these compounds

The majority of the identified ten compounds fell into the category of DNA damage-inducing agents, and also the RNAPI inhibitor CX-5461 is known to induce replication stress and DNA damage [30, 31]. To identify direct inhibitors of ribosomal subunit maturation, the exclusion of DNA damage as the source of inhibition of ribosome production is an important criterion. DNA double-strand breaks (DSBs) trigger the phosphorylation of the histone variant H2AX at serine Ser139, producing its phosphorylated form γH2AX [79], which aids the recruitment of DNA repair proteins to damaged chromatin. Phosphorylated γH2AX is enriched in nuclear foci that can be visualized by immunofluorescence [80, 81]. To implement γH2AX staining as a counter assay for DNA damage-induced DSBs, we treated HeLa K cells for 6 h with 1 µM of the identified DNA damage-inducing agents daunorubicin, doxorubicin, and mitoxantrone. As further controls, we included the translation inhibitors silvestrol and emetine. Etoposide was used as a positive control, as it induces DNA damage by inhibiting DNA topoisomerase II. As expected, immunofluorescence analysis with antibodies specific for γH2AX confirmed that the ribosome biogenesis defects induced by treatment of cells with daunorubicin, doxorubicin, or mitoxantrone were accompanied by severe DNA damage (Fig. 5A, B).

In order to quickly assess whether the identified compounds inhibit the proteasome, we developed an assay in which we exploited proteasomal degradation of a tetracycline-inducible fluorescent nuclear reporter protein carrying an auxin-inducible degron (AID). The AID system relies on the stable expression of the auxin-perceptive plant F-box protein TIR1, which forms a functional SCF^TIR1^ ubiquitin E3 ligase complex with human Skp1 and Cullin-1. Upon addition of the plant hormone auxin, proteins fused with a miniAID-tag (mAID) are recognized and ubiquitinated by SCF^TIR1^ and rapidly degraded by the proteasome [82, 83]. Our protein degradation reporter is composed of a nuclear localization signal (NLS), an HA epitope, a miniAID-tag, and a C-terminal eGFP and is expressed in HeLa K cells carrying a transgene encoding for OsTIR1-9myc. While the NLS-HA-mAID-eGFP reporter should be efficiently degraded in the presence of auxin under control conditions, inhibition of the proteasome is expected to inhibit its auxin-induced turnover. To evaluate the assay, cells expressing the reporter construct were treated for a total of 6 h with 1 µM of bortezomib or carfilzomib, and for the last 4 h before fixation additionally with auxin. As a negative control, we included the translation inhibitor CHX. MG132 was used as a positive control. Confocal fluorescence microscopy and immunoblotting confirmed that auxin efficiently induced the degradation of NLS-HA-mAID-eGFP under control conditions, whereas the auxin-induced degradation was impaired upon treatment with the respective proteasome inhibitors as expected (Fig. 5C, D). Thus, the established experimental setup represents a suitable counter assay to monitor changes in proteasome activity. Notably, the same tetracycline-inducible reporter protein allows to monitor defects in protein synthesis, based on examination of the nuclear levels of the NLS-HA-mAID-eGFP upon tetracycline induction. As predicted, treatment of cells with the translation inhibitor CHX indeed reduced the levels of NLS-HA-mAID-eGFP (in absence of auxin). Taken together, we have not only established an efficient and scalable screening pipeline to identify ribosome synthesis inhibitors, but also convenient visual counter assays for DNA damage, protein degradation, and synthesis.

## Discussion

In this study, we have implemented an imaging-based screening workflow to identify chemical compounds as inhibitors of the RB pathway in human cancer cells. Altogether, we established the experimental conditions for four different readouts that allow for detection of compound-induced defects in nuclear steps of human ribosome synthesis. All readouts are perfectly suited to detect nucleolar defects in subunit production that are intimately linked to p53 induction [23–25]. The four readouts complement each other: the ENP1(w/o LMB) readout responds to defects in pre-rRNA transcription, the RPS2-YFP and ENP1(plus LMB) readouts allow to detect defects in nucleolar steps of 40S subunit biogenesis, and the RPL29-GFP reporter is suitable to identify compromised nuclear 60S subunit synthesis. Still, the four readouts are not mutually exclusive since early steps of ribosome synthesis are tightly interwoven [20]. Compared to a previously published method exploiting a constitutively expressed Halo-RPS9 fusion protein [46, 47], our method is more versatile, exploiting a suite of complementing readouts for both 40S and 60S biogenesis, also allowing the detection of defects beyond nucleolar steps of 40S subunit maturation.

Our pilot screens using a library of FDA-approved small molecules demonstrated the suitability, sensitivity, and robustness of the implemented screening pipelines, which obtained high z′ scores of larger than 0.5 during quality control. We were able to identify expected hits, including translation inhibitors, proteasome inhibitors, and DNA damage-inducing agents. The most prominent identified DNA damage-inducing agents were the “rubicins” daunorubicin, epirubicin, idarubicin, and doxorubicin. Rubicins block DNA replication by inhibition of topoisomerase I [84] and were shown to interfere with ribosomal subunit maturation, causing different patterns of pre-rRNA processing defects [17]. For example, idarubicin inhibits pre-60S subunit maturation, whereas doxorubicin was shown to block rRNA transcription [16, 19]. Identification of these expected compounds as hits in our pilot screen provides further proof of the suitability of our screening assay.

The established assays are easily scalable and can be exploited for chemical compound libraries of far larger complexity. Our previous genome-wide RNAi screening campaigns have demonstrated that imaging capacity is not limiting for screens with many more assay plates. The subsequent computational analysis by supervised machine learning is also suited for far larger datasets [48–50]. Furthermore, the established assays can also be adapted to other cell types. The ENP1 immunofluorescence readout, in particular, is very versatile as it is readily applicable to any other cultured human cell line. For the use of the fluorescent ribosomal protein reporters, the corresponding tetracycline-inducible genes can be introduced by recombination into other cell lines carrying flippase recognition target (FRT) sites along with a Flp-recombinase expression vector or cloned into vectors allowing retroviral transduction.

Since the obtained hit compounds are known to target cellular pathways that indirectly affect ribosome biogenesis, we used them as proof-of-principle to establish adequate counter assays. In order to test whether candidate compounds elicit DNA damage, we detected the formation of nuclear γH2AX foci as a visual readout for DNA damage [80]. Expectedly, daunorubicin, etoposide, doxorubicin, and mitoxantrone treatment led to striking γH2AX foci formation. Interestingly, even though mycophenolate mofetil was shown to strongly induce DNA damage in rat bone marrow [85], our counter assays did not reveal increased γH2AX levels upon mycophenolate mofetil treatment. According to the mode of action of mycophenolate mofetil, ribosome synthesis is most likely inhibited by shortage of guanine nucleotides [68]. In addition, we established a counter assay that allows monitoring of proteasomal activity by exploiting a reporter protein with a degron for auxin-induced protein degradation.

The fact that various oncogenic pathways are linked to ribosome biogenesis and that some chemotherapeutic drugs are mechanistically connected to different ribosome maturation stages have strongly increased the interest in identifying inhibitors of ribosome biogenesis for development of new lead compounds. The phenotypic screening assays, together with the counter assays established here, provide a broad starting approach for compound identification and are expected to enable the rapid identification of novel, specific inhibitors of human ribosome synthesis by high-content screening. To date, this general approach is greatly complemented by a plethora of orthogonal methods that allow for subsequent target identification. These include chemical probe-based proteomics methods like photoaffinity labeling and affinity purification approaches, label-free methods such as chemical, proteolytic, or thermal stability profiling, or compound resistance screening [86–88]. In the future, AI-based target prediction might further expand this toolkit.

## Conclusions

To enable the identification of chemical compounds that affect nuclear steps of ribosome synthesis, we have established assays to perform single-cell, imaging-based screening campaigns. Exploiting distinct complementary readouts, we successfully completed pilot screens with more than 1000 FDA-approved drugs. These screens obtained excellent quality scores and identified altogether ten compounds as hits, which were used to establish counter assays for pathways that indirectly influence ribosome synthesis. Our screening pipelines provide a robust, efficient, scalable, and sensitive experimental framework for future identification of chemical compounds that impair ribosome synthesis.

## Methods

### Cell lines, cell culture and molecular cloning

All cell lines generated and used in this study were cultured in DMEM supplemented with 10% fetal bovine serum (FCS) and 100 µg/ml penicillin/streptomycin (DMEM +/+). Cells were grown at 37 °C in 5% CO_2_. HeLa K cells were a kind gift of D. Gerlich (IMBA, Vienna, Austria). The tetracycline-inducible RPL29-GFP and RPS2-YFP HeLa cell lines have been previously described [48, 49].

The reporter cell lines expressing RPS2-YFP were induced with 0.5 µg/ml tetracycline (Sigma, T7660) for 10 h, whereas the reporter cell lines expressing RPL29-GFP were induced for 8 h followed by a 10-h chase in tetracycline-free medium, with drug treatment for the last 6 h before the cells were fixed. Where indicated, HeLa K cells were treated with 20 nM leptomycin B (LC Laboratories) 90 min before fixation.

For random integration of OsTIR1-9myc into HeLa cells, a pIRESneo3 plasmid carrying the transgene was linearized by restriction digest with PvuI and subsequently transfected. Subsequently, cells were expanded, incubated, and selected with 400 µg/ml G418 for approximately 2 weeks until single colonies were visible. Single clones were picked and screened for expression levels and homogeneity by immunofluorescence and western blot analysis. To generate HeLa cells carrying a transgene for the inducible expression of the HA-mAID-NLS-eGFP fusion protein, the respective expression cassette was cloned into pcDNA5-FRT/TO carrying a hygromycin resistance gene and introduced into the HeLa cell lines by Flp-In recombination. Cells were transfected with 0.9 µg pOG44 carrying Flp recombinase and 0.1 µg of the pcDNA5-FRT/TO HA-mAID-NLS-eGFP plasmid. Twenty-four hours after transfection, cells were expanded and subjected to hygromycin B selection (0.3 mg/ml, Life Technologies Europe, 10,687,010). Single clones were picked and screened for expression of the transgene by immunoblotting and immunofluorescence. For expression of HA-mAID-NLS-eGFP, cells were induced with 0.5 µg/ml tetracycline for 24 h before auxin treatment (0.5 mM, 6 h, Sigma, I5148).

### Chemical compounds

For compound screening, we used the FDA-approved drug library from SelleckChem comprising 1172 small molecules. DMSO (Merck, 276,855), which served as solvent control, and six different positive controls were placed in columns 1, 2, 23, and 24 of each 384-well plate. The screening library and the controls were distributed over 4 master assay plates. For each of the compounds, 1 µl of a 0.6 mM compound solution in DMSO was spotted into deep-well plates and resuspended with 199 µl DMEM +/+ using an EL406 microplate washer dispenser (BioTek) on the day of treatment. Twenty-five microliters of the diluted compounds was added to the cells (in 50 µl DMEM +/+) using a LiquidatorTM 96-channel benchtop pipettor (Rainin) to yield a final drug concentration of 1 µM, and a final concentration of 0.17% DMSO. The following drugs were used as positive controls: actinomycin D (10 nM; Sigma Aldrich, A9415), bortezomib (2 µM; LC Laboratories, B-1408), CX-5461 (2.5 nM; SelleckChem, S2684), cycloheximide (100 µg/ml; Sigma Aldrich, C7698), MG132 (10 µM; Sigma Aldrich, C2211), and silvestrol (1 µM, MedChemExpress, HY-13251). For each of the four readouts, screening was performed in three biological replicates.

Etoposide (10 µM, Selleckchem, S1225) was used as a positive control in the counter assay for DNA damage. The following drugs were identified as hit compounds and tested in the counter assays: emetine (1 µM; Sigma Aldrich, E2375), carfilzomib (1 µM; Sigma Aldrich, 791,938), daunorubicin (1 µM; Sigma Aldrich, 30,450), mycophenolate mofetil (1 µM, Sigma Aldrich, SML0284), mitoxantrone (1 µM; Sigma, M6545), and doxorubicin (1 µM, Sigma Aldrich, D1515).

### Screening procedure

For the “ −/+ LMB ENP1” readout, HeLa K cells were detached with PBS/EDTA, counted, and mixed with DMEM +/+. For cell seeding, a suspension of 41,000 cells/ml was placed in a Wheaton spinning flask and put in a water bath at 37 °C. To avoid edge effects, due to temperature differences, the 384-well plates were placed on pre-warmed 37 °C metal blocks. Twenty-five microliters of HeLa K cells in DMEM +/+ (~ 2000 cells) was seeded per well and cultured overnight. The next day, cells were treated with the library compounds for 6 h. 4.5 h after drug treatment, cells were either treated with 10 µl LMB (“ + LMB ENP1,” final concentration: 20 nM) or 10 µl DMEM +/+ (“ − LMB ENP1”). Cells were then cultured for another 1.5 h and fixed with 4% formaldehyde after removing the medium. After 15-min incubation with 4% formaldehyde, plates were washed three times with 1 × PBS. Immunofluorescence for ENP1 was performed as described below with the following adjustments: Cell were permeabilized with 17 µl of detergent solution (0.1% Triton X-100, 0.02% SDS in 1 × PBS) for 5 min, followed by one wash with PBS. Blocking was performed with pre-blocking solution (2% BSA in 1 × PBS) for 30 min. Primary antibodies for ENP1 were diluted 1:12,500 and incubated in 10 µl in pre-blocking solution for 1 h, followed by four PBS washes. Secondary antibodies were diluted 1:300 and incubated for 30 min in pre-blocking solution, followed by four PBS washes. Plates were sealed and stored at 4 °C until imaging.

The reporter cell lines RPL29-GFP and RPS2-YFP were as described above and incubated overnight. Suspensions of 47,500 cells/ml of HeLa RPL29-GFP (40 µl/well) or 30,000 cells/ml HeLa RPS2-YFP (40 µl/well) were used. The next day, the HeLa RPL29-GFP cells were induced with 10 µl tetracycline (final concentration 0.5 µg/ml) and incubated for 8 h. Cells were then washed with DMEM −/− and cultured in tetracycline-free DMEM +/+ for 10 h. At the same time, HeLa RPS-YFP cells were induced with 10 µl tetracycline (final concentration 0.5 µg/ml) and incubated for 10 h. Six hours before fixation, cells were treated with the library compounds as described above. Cells were fixed for 15 min at room temperature (RT) with 4% formaldehyde containing 1 µg/ml Hoechst, washed three times with 1 × PBS, and the plates sealed with foil. Plates were stored at 4 °C overnight before imaging to assure optimal distribution of Hoechst staining in the nuclei. Plates were warmed up to RT and imaged on a Molecular Devices Image Xpress microscope with a 10 × objective (3 × 3 images/well). Laser-based autofocus was performed at each site. Image acquisition of channels was performed sequentially.

### Image analysis, phenotypic cell classification by machine learning and hit list generation

Images were corrected for illumination problems (also called vignetting effect), which is an inherent optical error of any imaging system. To correct this effect, we used the CIDRE method [89]. CIDRE automatically learns two functions, the 0th order additive term that eliminates the camera sensor’s effect and the 1 st order term that corrects for the optical distortion. This correction resulted in an image set that is as close to real fluorescence signals as possible. Images were not corrected for out-of-focus, but those with focus problems were excluded from the further analysis. Cells were segmented based on the Hoechst staining of nuclei and feature extraction was performed using CellProfiler [63]. The cytoplasm was estimated by adding a ring around the segmented nucleus (width of 6.5 µm), and nuclei and cytoplasm were used as masks to extract a variety of cell features. Classification of cells based on their phenotypes was performed using Advanced Cell Profiler (ACC) [62]. In total, eight classes of cells were defined based on the localization of the reporter proteins and cell features: nucleolar, nucleolar/nucleoplasmic, nucleoplasmic, cytoplasmic, mitotic cells, apoptotic cells, cells without fluorescence signal, and incorrectly segmented cells. Cells were classified using the Weka setting, and hit rates and z′ scores were calculated for each compound and control. For hit list generation, a threshold was set by adding five standard deviations to the mean of the DMSO control. Hits for the different readouts were defined in the following manner:

**Table:** 

Readout	Hit	Non-hit
− LMB ENP1	Nucleoplasmic signal	Nucleolar signal, nucleolar/nucleoplasmic signal
+ LMB ENP1	Nucleolar signal Nucleolar/nucleoplasmic signal	Nucleoplasmic signal
RPL29-GFP	Nucleolar signal Nucleolar/nucleoplasmic signal Nucleoplasmic signal	Cytoplasmic signal
RPS2-YFP	Nucleolar signal Nucleolar/nucleoplasmic signal Nucleoplasmic signal	Cytoplasmic signal

For all readouts, hit rates were determined by dividing the number of hits by the sum of hits and non-hits.

### Immunofluorescence and confocal microscopy

For follow-up analyses, cells were grown on coverslips and fixed with 4% formaldehyde for 12–15 min at RT and washed 3 times with 1 × PBS. For immunofluorescence analysis, cells were permeabilized in 1 × detergent solution (0.1% Triton X-100, 0.02% SDS in 1 × PBS) for 5 min and transferred to blocking solution (2% BSA in 1 × PBS, 10% goat serum). Cells were incubated in blocking solution for at least 30 min at RT followed by 1-h incubation at RT in primary antibodies diluted in blocking solution. Coverslips were washed three times with 2% BSA for 4 min each and subsequently incubated in secondary antibodies, including 1 µg/ml Hoechst, for 30 min at RT. Cells were washed three times with 1 × PBS, incubated for 1 min with detergent solution, and fixed for 5 min with 4% formaldehyde. Coverslips were mounted on glass slides using Vectashield and sealed with nail polish. The following primary antibodies were used in this study: anti-HA 3F10 (rat, Roche 11,867,423,001), anti-HSP60 (rabbit, Abcam, 45,134), anti-γH2AX (mouse, Millipore, 05–636-I). The α-rabbit ENP1 antibody has been described previously [45]. The following secondary antibodies were used in this study: α-mouse Alexa Fluor Plus 680 (Thermo Fisher Scientific, A32729), α-rabbit Alexa Fluor Plus 800 (A32735), α-mouse Alexa Fluor 594 (A-11005), α-rabbit Alexa Fluor 488 (A-11008), α-rat Alexa Fluor 488 (A-11006).

Microscopy was performed at the Scientific Center for Optical and Electron Microscopy (ScopeM) at ETH Zurich. Immunofluorescence samples were imaged with an Olympus FluoView 300 confocal scanning system using a 60 ×, 1.3 NA, silicone oil, super apochromat (SAPO) objective. Fiji and Adobe Photoshop were used for image processing. For high-throughput screening of the assay plates generated during the pilot screens, the Molecular Devices Image Xpress microscope with a 10 × objective was used.

### Counter assays

To visualize DNA damage induced by the candidate compounds, HeLa K cells were treated for 6 h with 1 µM of the hit compounds daunorubicin, doxorubicin, or mitoxantrone. Etoposide (10 µM) was used as a positive control; silvestrol (1 µM), actinomycin D (10 nM), emetine (1 µM), and mycophenolate mofetil (1 µM) were used as negative controls. Immunofluorescence analysis using antibodies specific for γH2AX allowed for visualization of DNA damage by DSBs. Inhibition of proteasomal degradation was analyzed by exploiting the auxin-inducible degron system. Cells expressing a tetracycline-inducible HA-mAID-NLS-eGFP transgene were treated for 6 h with 1 µM of the candidate compounds. 0.5 mM auxin (indole-3-acetic acid) was added 4 h before fixation. Confocal fluorescence microscopy and immunoblotting were exploited to visualize the levels of the HA-mAID-NLS-eGFP construct. For quantification of the levels of HA-mAID-NLS-eGFP, microscopy images were analyzed with Cell Profiler [63]. Nuclei were segmented using the DAPI signal and the integrated GFP signal intensity of each nucleus was determined.

### Northern blotting

For Northern blot analysis, between 1.5 and 2 µg total RNA were extracted from HeLa cells, mixed 1:1 with 2 × FLB buffer (40 mM HEPES pH 7.8, 36% (v/v) formamide, 12% (v/v) formaldehyde, 20% (v/v) glycerol, 0.05% xylene cyanol, 0.05% bromophenol blue, GelRed), and incubated at 95 °C for 5 min. Samples were separated by agarose gel electrophoresis (1.2% gel) containing 6% formaldehyde. A loading control image was taken on a GelDox XR + (Bio-Rad) system. The gel was subsequently washed and incubated with the following solutions: 15 min in 75 mM NaOH, 20 min in 0.5 M Tris pH 7.0, 1.5 M NaCl, and 15 min in 10 × SSC. The RNA was transferred onto a nylon membrane (Hybond-N +, GE Healthcare) by capillary transfer for 72 h at room temperature. RNA was cross-linked by three rounds of UV exposure (120 mJ/cm^2^) using an UV Stratalinker (Stratagene). The membrane was incubated in pre-hybridization buffer for 1 h at 65 °C. The ITS1 probe (CCTCGCCCTCCGGGCTCCGTTAATGATC) was radioactively labeled with [γ−32P]-ATP using T4 PNK at 37 °C for 1 h and cleaned using a MicroSpin G-50 column (GE Healthcare) according to manufacturer’s instructions. The probe was added to the membranes in pre-hybridization buffer (50% (v/v) formamide, 5 × SSPE, 5 × Denhardt’s solution, 1% SDS, 200 µg/ml DNA) and incubated for 1 h at 65 °C, and then at 37 °C overnight. The membrane was washed three times in 15 ml SSC. The membrane was wrapped in plastic and analyzed by phosphoimaging (Typhoon FLA 900 imager, GE Healthcare).

## Supplementary Information

Additional file 1: Fig. S1. Effect of established inhibitors on the four screening readouts. Fig. S2. Analysis of the effect of mycophenolic acid and mycophenolate mofetil on 18S pre-rRNA processing and 40S subunit biogenesis.

Additional file 2: Table S1. Full numerical screening data of all screening replicates.

Additional file 3: Table S2. Numerical data of the analysis of the screening controls for all replicates, used for Fig. 3.

Additional file 4. Uncropped images of blots.

## Data Availability

The generated datasets generated are contained in the manuscript except for the raw microscopy data of the screens. Images are available in the BioImage Archive (https://www.ebi.ac.uk/bioimage-archive/ accession: S-BIAD2153,  https://www.ebi.ac.uk/biostudies/bioimages/studies/S-BIAD2153) which are available at the EMBL-EBI BioImage Archive (https://www.ebi.ac.uk/bioimage-archive/) [90].

## Abbreviations

RPs: Ribosomal proteins
SSU: Small subunit
RNAPI: RNA polymerase I
rRNA: Ribosomal RNA
RNAi: RNA interference
LMB: Leptomycin B
