# Supplementary material for: Establishment of an imaging-based screening pipeline for the identification of human ribosome biogenesis inhibitors
Source: BMC Biol. 2025 Oct 21;23:315. doi: 10.1186/s12915-025-02425-2 (PMC12542422; doi:10.1186/s12915-025-02425-2)
Supplement: Supplementary file 1 — Additional file 1: Fig. S1. Effect of established inhibitors on the four screening readouts. Fig. S2. Analysis of the effect of mycophenolic acid and mycophenolate mofetil on 18S pre-rRNA processing and 40S subunit biogenesis. [file 12915_2025_2425_MOESM1_ESM.pdf]

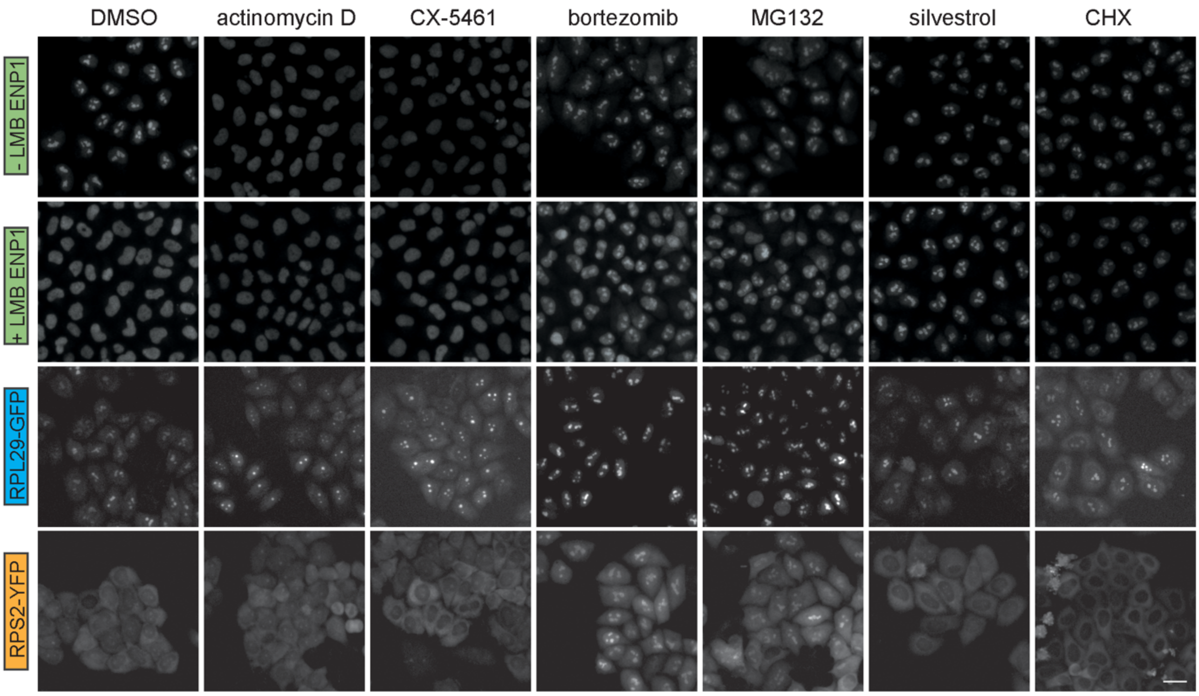

Suppl. Fig. 1 Analysis of the response of the four readouts to established inhibitors of RNAPI transcription (ActD, CX-5461) or compounds that perturb ribosome synthesis indirectly by affecting the proteasome (bortezomib, MG132) or mRNA translation (silvestrol, CHX).

Reporter expression (RPL29-GFP; RPS2-YFP) was induced with 0.5 µg/ml tetracycline as described in Fig. 1. Cells were treated with the indicated compounds (10 nM ActD, 2 nM CX-5461, 2 µM bortezomib, 10 µM MG132, 1 µM silvestrol, 100 µg/ml CHX) for 6 h. HeLa K cell were treated with 10 nM leptomycin B (LMB) for 90 min before cell were fixed (for +LMB ENP1 immunofluorescence). Cells were analyzed by confocal microscopy. Scale bar: 20 µm.

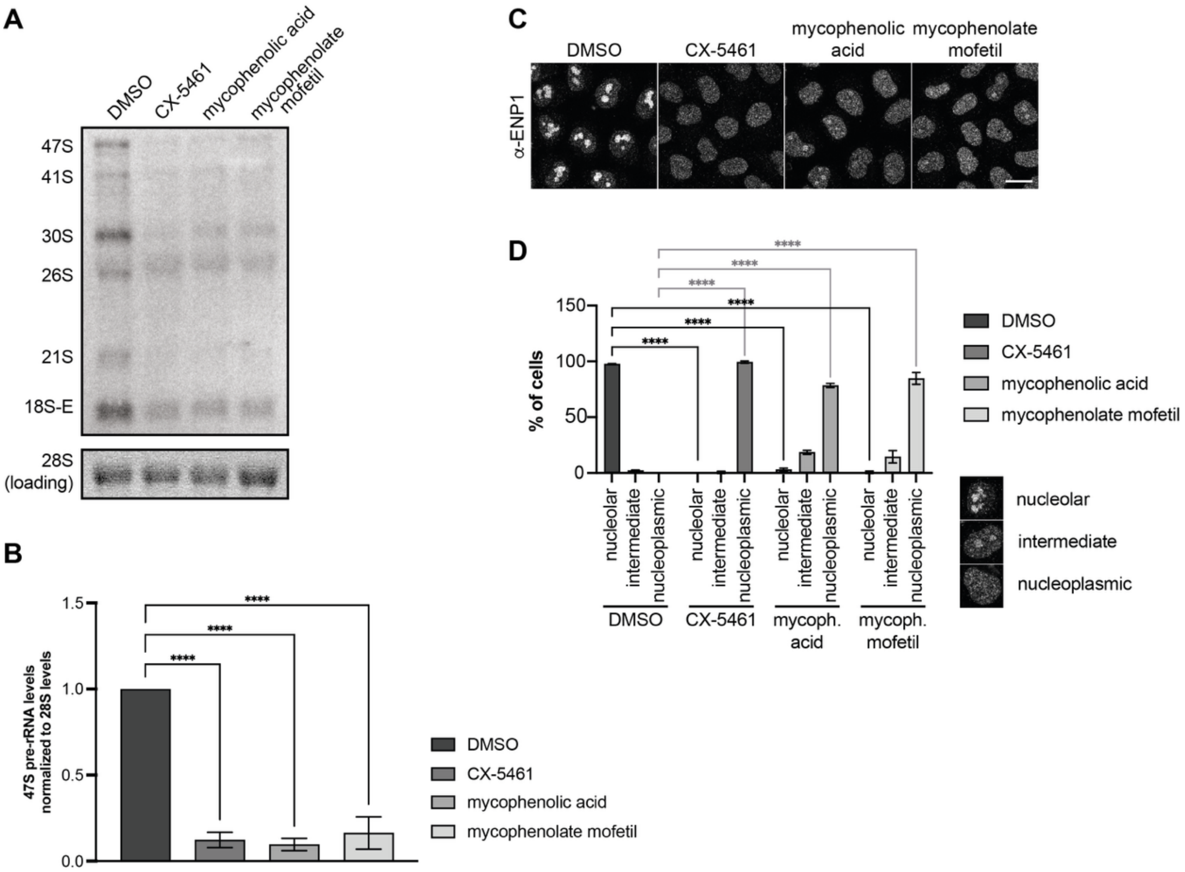

Suppl. Fig. 2 Analysis of the effect of mycophenolic acid and mycophenolate mofetil on 18S pre-rRNA processing and 40S subunit biogenesis.

A HeLa cells were treated with 1 µM of the indicated compounds or the solvent control DMSO for 6 h. Total RNA was extracted, separated by agarose gel electrophoresis in presence of GelRed (28S RNA loading control), transferred to a nylon membrane, and analyzed by Northern blotting using a probe directed to the internal transcribed spacer 1 (ITS1), which labels precursors of 18S rRNA.

B Quantification of 47S pre-rRNA in **A** relative to the 28S RNA loading control and normalized to DMSO. Mean -/+ SEM, N = 3, one-way Anova, ****$p \leq 0.0001$.

C HeLa cells were treated as in A, fixed and analyzed by immunofluorescence (confocal microscopy) using an anti-ENP1 antibody. Scale bar: 20 µm.

D Quantification of ENP1 localization in cells in **C**. Cells were manually assigned to the indicated categories and counted. Mean -/+ SEM, N = 3, between 101 and 128 cells counted per replicate and condition, two-way Anova, ****$p \leq 0.0001$ (in black for nucleolar localization, in grey for nucleoplasmic localization).
