# Supplementary material for: Establishment of an imaging-based screening pipeline for the identification of human ribosome biogenesis inhibitors
Source: BMC Biol. 2025 Oct 21;23:315. doi: 10.1186/s12915-025-02425-2 (PMC12542422; doi:10.1186/s12915-025-02425-2)

Gafko et al., Establishment of an imaging-based screening pipeline for the identification of human ribosome biogenesis inhibitors: uncropped gel/blots

Dotted boxes contain the data shown in the Figure of the manuscript.

for Figure 5E: uncropped Western blots

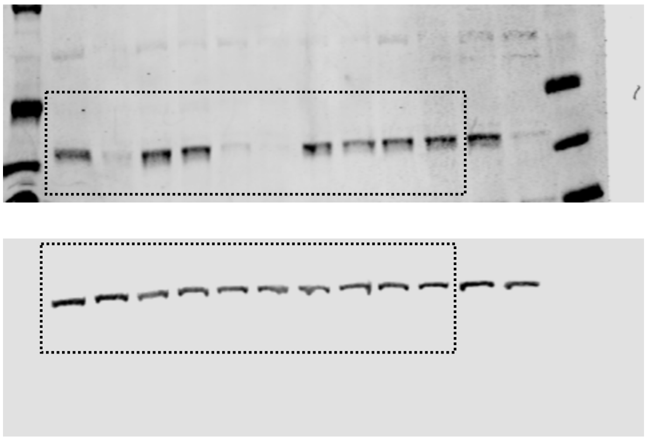

for Supplemental Figure S2: uncropped Northern blot and GelRed-stained membrane (RNA loading)

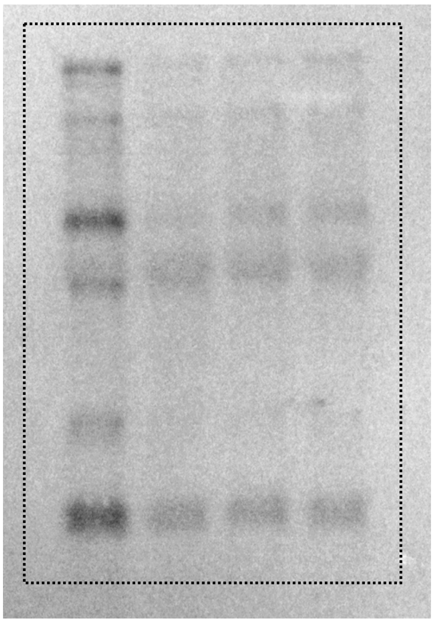

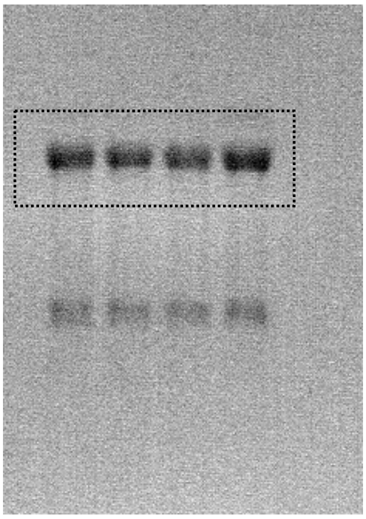

Supplement: Supplementary file 4 — Additional file 4. Uncropped images of blots. [file 12915_2025_2425_MOESM4_ESM.pdf]
